# Discrimination and Oral Health Impact: Moderating Role of Sex and Sexuality

**DOI:** 10.1177/00220345241310223

**Published:** 2025-02-14

**Authors:** G.H. Soares, S. Sethi, A. Jessani, L. Jamieson

**Affiliations:** 1 Australian Research Centre for Population Oral Health, Adelaide Dental School, University of Adelaide, Adelaide, Australia; 2 Schulich School of Medicine and Dentistry, Western University, London, Canada

**Keywords:** adolescents, sexual and gender minorities, LGBTQ, gender diverse, dental caries, quality of life

## Abstract

This study investigated whether sex assigned at birth and sexuality have a moderating role on the effect of discrimination on oral health impacts among adolescents. Using data from the Longitudinal Study of Australian Children, a sample representative of all Australian adolescents aged 14 to 15 y (*N* = 2,905), we employed propensity score overlap weights to achieve covariate balance between participants exposed and unexposed to discrimination. Various forms of discrimination were examined: due to cultural background, due to mental health condition, due to sexual orientation, and due to sex assigned at birth. Oral health impact was assessed using the PedsQ Oral Health Scale. Poisson regression with robust variance was conducted including sex, sexual attraction, and discrimination as interaction terms. The sample included 1,407 females (49%) and 336 lesbian, gay, bisexual, and questioning (LGBQ) individuals (11%). More than 22% experienced at least 1 form of discrimination in the previous 6 mo. Findings from the overlap weighting analysis revealed that, in general, females had higher proportions of oral health impact compared with males, whereas sexually diverse youth tended to have worse oral health outcomes compared with non-LGBQ youth. A 2-fold higher prevalence rate of oral health impacts was found for sexually diverse females exposed to discrimination due to cultural background (95% confidence interval [CI]: 1.36–2.44) and due to mental health conditions (95% CI: 1.62–2.46). The largest effects of discrimination on oral health impacts were consistently observed among sexually diverse females. This novel study provides evidence on the moderating role of sex and sexuality in the relationship between discrimination and oral health among adolescents.

## Introduction

Discrimination is characterized by the sustained, differential, and unjust treatment of groups or individuals on the basis of any measure of social diversity (Bhugra 2016). It not only affects the lives of marginalized populations but also harms society at large by violating the basic human rights principle of equality. Discrimination must be understood as a manifestation of structural systems of oppression that generate unfair distributions of power, resources, and opportunities. These structures include colonialism, racism, sexism, homophobia, and transphobia, which are often interconnected. Discrimination targeting stigmatized populations is usually perpetrated at both interpersonal and institutional levels, affecting key social determinants of health such as housing, employment, and access to health care (Beltran et al. 2019; Williams et al. 2019; Lindsay et al. 2023).

The compound effects of discriminatory practices have been acknowledged in structuring social injustices and oral health inequities (Bastos et al. 2022). At a structural level, discrimination can harm oral health through systematically limiting peoples’ access to power, prestige, freedom, and social gains (Bastos et al. 2020). The effects of discrimination on dental outcomes can be illustrated by the persisting racial inequities in oral health. Bastos et al. (2022) reported that structural racism and sexism are associated with a 60% increased odds of edentulism for Black individuals. At a proximal level, social stress theory posits that experiences of discrimination are a significant stressor that can promote a cascade of negative effects on health (Hatzenbuehler and Pachankis 2016). Research has shown the impact of perceived discrimination on neural functioning, psychological well-being, and psychiatric disorders (Clark et al. 2018). Coping with chronic distress can further drive biological dysregulation and contribute to adverse health behaviors, including reduced engagement with health services (Williams et al. 2019).

The impact of discrimination on adolescents can have negative repercussions that extend well into adulthood (Birkett et al. 2015). Victimization (i.e., acts in which an individual or group of individuals are subject to cruel or unjust treatment) represents a ubiquitous issue across schools (Richardson and Hiu 2018). Identity markers such as ethnicity, disability status, and mental health intersect with sex and sexuality, creating complex layers of discrimination for minority youth. In addition to experiencing homophobia, sexually diverse youth from racialized communities are frequently exposed to racism (Le et al. 2022). Boys and girls experience victimization differently based on how they express gender stereotypes associated with ideals of masculinity and femininity (Wright and Wachs 2020). Sexually diverse youth—and particularly sexually diverse girls—are at an increased risk of suicidal feelings and self-harm as a result of bullying, family rejection, and violence (Australian Institute of Health and Welfare 2023; Jadva et al 2023).

While there is an emerging body of literature articulating the effects of racism on the oral health of racialized groups (Bastos et al. 2018; Bastos et al. 2020; Noronha et al. 2023), evidence on the relationship between oral health and other forms of discrimination is scarce (Bastos et al. 2022). Studies investigating gender- and sexuality-based oral health disparities have overlooked the role of discrimination in creating these inequities (Schwartz et al. 2019; Gupta et al. 2023). In the present study, we address the largely unexplored question of how various forms of discrimination affect the oral health of adolescents based on sex and sexual attraction. The effects of discrimination due to cultural background, mental health, sexual discrimination, and sex were considered. Specifically, the aim of this study was to investigate the moderating role of sex and sexual attraction on the effect of discrimination on oral health impacts among adolescents.

## Methods

### Data source

This is an ancillary study of the Longitudinal Study of Australian Children (LSAC), a sample representative of all Australian children born between March 2003 and February 2004 (Schwartz et al. 2019). Cross-sectional data were obtained from the B cohort (wave 8). The study adopted a 2-stage random sampling strategy using Australian postal codes as the primary sampling unit. The initial sample comprised 5,107 infants aged 0 to 1 y. Wave 8 was conducted in 2018 and generated information on 3,127 participants aged 14 to 15 y. Self-report data were generated through survey questions answered by the study participant and administered by an interviewer. Ethics approval for the LSAC study was granted by the Australian Institute of Family Studies Ethics Committee (application No. 17-01). The research team obtained permission from the Australian Data Archive Dataverse to use LSAC data for the purposes outlined in this study (ref. 274601). A confidentiality deed was signed, and anonymized data were used for the analysis. The reporting of this study adheres to the STROBE statement.

### Outcome

Oral health impact was assessed using the self-report version of the PedsQ™ Oral Health Scale (Steele et al. 2009). Participants were asked to report the frequency of 5 oral health problems: (1) tooth pain; (2) tooth pain when eating or drinking something cold, hot, or sweet; (3) teeth that are dark in color; (4) gum pain; and (5) blood on the toothbrush after brushing. Responses were recorded using a 5-point scale. Responses recorded as “never” and “almost never” (codes 0 and 1) were classified as “no oral health impact,” whereas responses rated as “sometimes,” “often,” and “almost always” (codes 2, 3, and 4) were classified as “presence of oral health impact.” A binary outcome variable was created by combining the number of items with the presence of oral health impact and categorizing participants into 2 groups: no oral health impact versus 1 or more oral health impacts.

### Sexual Attraction and Sex Assigned at Birth

Sexual attraction was measured based on adolescents’ self-reported sexual attraction. Sexual attraction is an appropriate measure of sexual orientation for the developmental stage of adolescence. In contrast to domains of sexual behavior and sexual identity, attraction often takes place as the central viewpoint through which adolescents understand their sexuality (Friedman et al. 2004). Adolescents attracted to the same sex, both sexes, or unsure about their attraction were grouped into a sexuality diverse category including lesbian, gay, bisexual, and questioning individuals (LGBQ). Adolescents expressing attraction to members of the opposite sex only and adolescents expressing no attractions were classified as non–sexuality diverse youth (non-LGBQ). It is important to note that adolescents may experience sexual attraction at different stages of development. For this reason, adolescents who expressed no attraction were classified as “non-LGBQ.” Throughout this study, the terms *LGBQ* and *sexually diverse* were used interchangeably to refer to study participants who express nonheterosexual orientations or experiences. The adopted terminology does not include gender-diverse individuals. Sex assigned at birth was recorded at baseline as a binary variable (male or female).

### Experience of Discrimination

Different forms of discrimination were investigated: (1) cultural background discrimination, (2) discrimination due to mental health, (3) discrimination due to sexual orientation, and (4) discrimination due to sex. Experience of cultural background discrimination was assessed by asking adolescents whether in the past 6 mo they had been treated unfairly or badly because of their language or accent, skin color, religious beliefs, or cultural background. Participants were also asked whether in the past 6 mo they had been treated unfairly or badly due to mental health problems, sexual identity or sexual orientation, and due to sex. Responses to each form of discrimination were recorded as yes/no.

### Confounders

Potential confounders included factors likely to influence both oral health impact and experience of discrimination: age (in months), self-rated general health, special care needs (whether the participant requires extra medical care, mental health, or educational services), weekly household income, area-level index of relative socioeconomic advantage and disadvantage, and geographic remoteness. Parents provided information on special care needs and income through a structured questionnaire. Information on self-rated general health was provided by the study participants.

### Propensity Score Overlap Weighting

We used propensity score (PS) overlap weighting to adjust for confounding between experience of discrimination and oral health impact (Li et al. 2019). First, multivariable logistic regression models were built to estimate PSs. To generate overlap weights, participants exposed to discrimination were weighted by the probability of being unexposed (1-PS), whereas participants unexposed to discrimination were weighted by the probability of being exposed (PS) (Thomas et al. 2020). This method up-weights participants whose characteristics are compatible with either treatment (exposed/unexposed) while reducing the influence of outliers with extreme PSs. Participants at the ends of the PS distribution (with very high or very low probabilities of being assigned to a given treatment) receive proportionally smaller weights. PS overlap weighting overcomes limitations of other weighting methods by improving precision and balance without excluding any participants. It provides exact balance on the mean of all measured covariates when estimated using logistic models (Li et al. 2018). This technique results in a pseudo-population that mirrors a pragmatic randomized trial in which no participants are removed from the sample.

Survey weights were applied to the logistic models used to estimate PS. Overlap weights were constructed separately for each level of the moderator variable (1 = male non-LGBQ; 2 = female non-LGBQ; 3 = male LGBQ; 4 = female LGBQ). In other words, we achieved exchangeability across measured confounders between individuals exposed and unexposed to discrimination, both within each category of the moderator and across the whole sample (Griffin et al. 2023). Standardized differences less than 0.1 (10%) were indicative of negligible imbalance between groups.

### Analytical Approach

The analytical sample included all participants with complete information on oral health impacts. Proportions of missing information on dependent variables and covariates were minimal, ranging from 0.0% to 2.6%. Analysis was conducted using the complete case sample (*N* = 2,905).

We investigated whether sex and sexual attraction moderate the effect of discrimination on oral health impact. Prevalence rate (PR) estimates and 95% confidence intervals (95% CIs) were derived from Poisson regression with robust variance including interaction terms between sex, sexual attraction, and discrimination. A separate model was built for each form of discrimination. Outcome models incorporated the product of overlap weights and survey weights. The analysis of discrimination related to sexual orientation was limited to LGBQ participants. We assessed whether sex moderates the association between sexual orientation discrimination and oral health impact among sexually diverse youth. Similarly, the effect of discrimination related to sex was investigated within the group of female participants. We examined whether sexual attraction moderated the effect of sex-related discrimination on oral health impact among females. Predicted probabilities were obtained from each model.

### Sensitivity Analysis

To assess the robustness of our findings, we examined how different weighting methods affected our estimates. We used a doubly robust estimator procedure to assess potential errors in model misspecification. Doubly robust outcome models were constructed including covariates from the PS model in addition to composite overlap weights (DROW). By combining the 2 approaches, the estimator only requires either of the 2 models to be correctly specified to obtain unbiased results. We also assessed the sensitivity of estimates via doubly robust inverse probability weights (DRIPW).

## Results

A total of 2,905 participants were included in the sample. Approximately 11% of the sample (*n* = 336) expressed sexually diverse orientations or experiences (Table 1). Approximately 49% (*n* = 1,407) had a female binary sex assigned at birth. More than 22% (95% CI: 20.5%–24%) (*n* = 638) experienced at least 1 form of discrimination in the past 6 mo. Discrimination due to cultural background had the highest prevalence (13.1%). Sexually diverse females had considerably higher proportions of experience of discrimination due to mental health and sex than their counterparts did. The overall prevalence of oral health impact was 43% (40.9%–45.1%). The prevalence of oral health impact ranged from 37.7% among non-LGBQ males to 60.6% among LGBQ females.

**Table 1.** Descriptive Characteristics of the Sample by Sexual Attraction and Sex.

	Total Sample (*N* = 2,905)		Non-LGBQ				LGBQ			
			Male (*n* = 1,392)		Female (*n* = 1,177)		Male (*n* = 106)		Female (*n* =230)	
Variable	*n*	% (95% CI)	*n*	% (95% CI)	*n*	% (95% CI)	*n*	% (95% CI)	*n*	% (95% CI)
Oral health impact (yes)	1,211	43.0 (40.9–45.1)	528	37.7 (34.8–40.8)	507	45.6 (42.3–49.0)	45	45.5 (35.2–56.3)	131	60.6 (53.3–67.3)
Age (mo)^a^		178.3 ± 3.9		178.3 ± 4.6		178.4 ± 4.6		178.3 ± 4.6		178.1 ± 4.6
Self-rated general health										
Excellent/very good	2,079	67.1 (62.0–71.9)	1,041	74.1 (71.4–76.7)	852	68.9 (65.6–72.0)	64	61.3 (50.6–71.1)	122	51.4 (44.0–58.7)
Good/fair/poor	826	32.9 (28.1–38.0)	351	25.9 (23.3–28.6)	325	31.1 (28.0–34.4)	42	38.7 (28.9–49.4)	108	48.6 (41.3–56.0)
Special care needs										
Yes	339	10.9 (8.4–14.1)	155	12.0 (10.0–14.2)	124	10.4 (8.6–12.7)	20	16.4 (10.5–24.7)	40	16.7 (12.0–22.8)
No	2,566	89.1 (85.9–91.6)	1,237	88.0 (85.8–90.0)	1,053	89.6 (87.3–91.4)	86	83.6 (75.3–89.5)	190	83.3 (77.2–88.0)
Remoteness										
Major cities	1,892	67.3 (62.4–71.9)	913	67.7 (65.0–70.3)	748	66.3 (63.3–69.1)	71	67.2 (56.9–76.1)	160	69.7 (62.6–76.0)
Regional	1,013	32.7 (28.1–37.6)	479	32.3 (29.7–35.0)	429	33.7 (30.9–36.7)	35	32.8 (23.9–43.1)	70	30.3 (24.0–37.4)
Weekly income (AU$)^a^		1,093.7 ± 868.0		1,057.3 ± 892.4		1,109.6 ± 942.2		1,269.8 ± 1,309.4		1,149.1 ± 950.4
SEIFA^a^		1,008.4 ± 77.5		1,009.2 ± 87.5		1,006.9 ± 91.9		1,013.7 ± 100.8		1,008.0 ± 100.8
Discrimination										
Cultural background	352	13.1 (11.7–14.7)	175	13.5 (11.4–15.8)	129	11.8 (9.8–14.3)	17	15.8 (9.6–24.9)	31	16.2 (10.9–23.5)
Mental health	183	6.6 (5.6–7.7)	53	4.1 (3.0–5.5)	83	7.7 (6.1–9.8)	7	5.3 (2.4–11.2)	40	16.9 (12.0–23.2)
Sexual orientation	94	3.3 (2.6–4.1)	15	1.1 (0.6–2.0)	10	0.9 (0.4–1.9)	22	18.5 (12.0–27.4)	47	22.1 (16.4–29.2)
Sex	163	5.7 (4.8–6.7)	19	1.3 (0.8–2.2)	98	8.5 (6.8–10.6)	5	5.5 (2.0–14.0)	41	18.0 (13.0–24.3)

a Age, weekly income, and Socio-Economic Indexes for Areas (SEIFA) are presented as mean and standard deviation.

**Figure 1.**
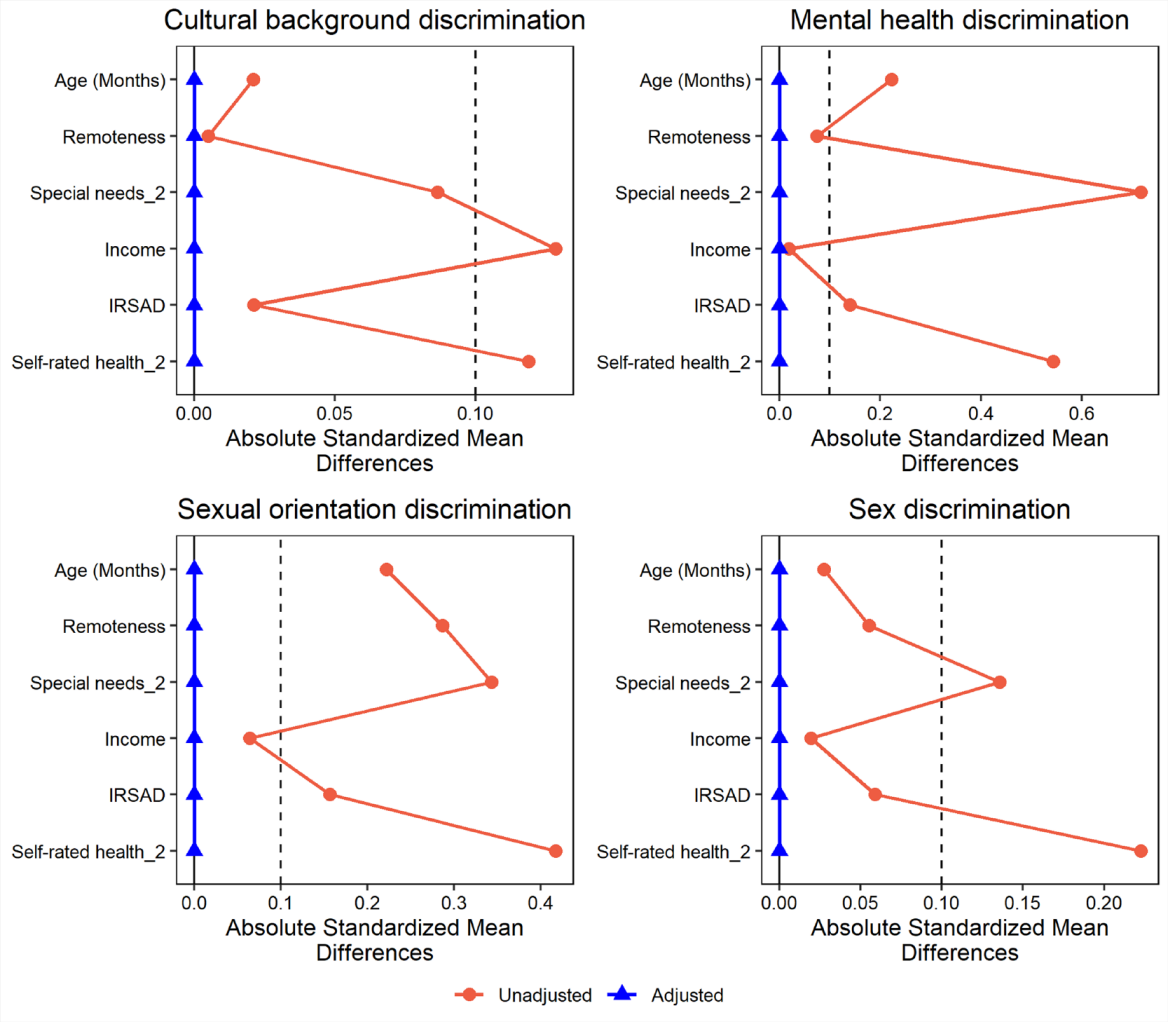
Standardized differences in covariates between adolescents exposed and unexposed to discrimination before and after application of composite weights (overlap weights multiplied by survey weights).

The exact balance on the mean of all measured covariates was obtained for all exposures when employing overlap weights (Fig. 1). Overlap weights also yielded exact covariate balance within all levels of the moderator variable (Appendix Figs. 1–4). In the DRIPW analysis, nonnegligible imbalance persisted within levels of the moderator for a number of variables.

Findings of the outcome models incorporating the product of overlap weights and survey weights are presented in Table 2. Overall, youth who experienced discrimination due to cultural background had a 19% higher PR of oral health impact (95% CI: 1.04–1.36). Discrimination due to mental health was associated with a 27% higher PR of oral health impact (95% CI: 1.09–1.48). The PR of oral health impact among LGBQ youth was 38% (95% CI: 1.13–1.68) among those who experienced discrimination due to sexual orientation. Among females, experience of discrimination due to sex was associated with a 27% higher PR of oral health impact (95% CI: 1.08–1.50).

**Table 2.** Estimated Effects of Discrimination on Prevalence of Oral Health Impact by Sexual Attraction and Sex.

	Non-LGBQ		LGBQ	
	Males	Females	Males	Females
Discrimination	PR (95% CI)	PR (95% CI)	PR (95% CI)	PR (95% CI)
Cultural background				
Absent	1 (Ref.)^a^	1.24 (1.1–1.4)	1.11 (0.79–1.57)	1.66 (1.39–1.93)
Present	1.22 (0.98–1.5)	1.44 (1.15–1.75)	1.39 (0.63–2.1)	1.92 (1.36–2.44)
Mental health				
Absent	1 (Ref.)^a^	1.24 (1.05–1.45)	1.33 (0.94–1.87)	1.49 (1.19–1.83)
Present	1.11 (0.77–1.6)	1.66 (1.32–2.04)	1 (0.36–2.77)	2.04 (1.62–2.46)
Sexual orientation				
Absent			1 (Ref.)^b^	1.24 (0.9–1.71)
Present			1.2 (0.74–1.96)	1.78 (1.34–2.52)
Sex				
Absent		1 (Ref.)^c^		1.31 (1.11–1.55)
Present		1.32 (1.07–1.62)		1.54 (1.16–1.92)

CI, confidence interval; LGBQ, lesbian, gay, bisexual, and questioning; PR, prevalence rate.

a Reference category: male non-LGBQ not exposed to discrimination.

b Reference category (sexual orientation model): male LGBQ not exposed to discrimination.

c Reference category (sex model): female non-LGBQ not exposed to discrimination.

**Figure 2.**
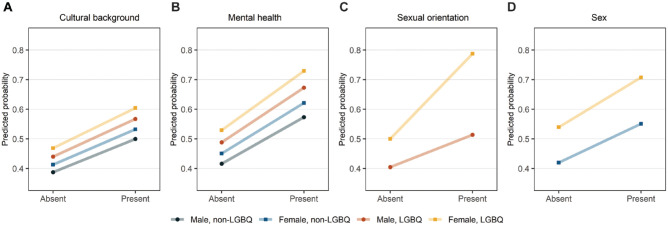
Predicted probabilities of oral health impact among adolescents exposed and unexposed to discrimination by sex and sexual attraction.

The effect of discrimination on oral health impact varied considerably across levels of the moderator. All groups with experience of discrimination had a higher PR of oral health impact, except for sexually diverse males in the mental health discrimination model. This is possibly due to a small sample size in this subgroup (*n* = 7). The largest effects of discrimination on oral health impact were consistently observed among sexually diverse females. Two-fold higher PRs of oral health impact were observed for sexually diverse females who reported discrimination due to cultural background (95% CI: 1.36–2.44) and due to mental health (95% CI: 1.62–2.46) compared with non-LGBQ males with no experience of discrimination. Experience of discrimination due to sexual orientation was associated with a 78% higher PR of oral health impact (95% CI: 1.34–2.52) among sexually diverse females compared with sexually diverse males. Discrimination due to sex was associated with a 54% increased PR of oral health impact (95% CI: 1.16–1.92) among sexually diverse females compared with non-LGBQ females. Predicted probabilities for each level of the moderator are presented in Figure 2.

### Sensitivity Analysis

Table 3 shows the estimated effect of discrimination on oral health impact across the entire sample when using different weighting strategies. The effects obtained using doubly robust estimators were consistent with the effects yielded using overlap weights. The doubly robust overlap weighting estimator yielded an 8% higher effect size for mental health discrimination compared to overlap weighting, the largest difference observed across all models. All models employing doubly robust estimators produced similar gradients across levels of the moderator, with the worst outcomes for LGBQ females exposed to discrimination (Appendix Table 6).

**Table 3.** Comparison of Estimated Effects according to Different Weighting Techniques.

Discrimination	Survey Weights	Overlap Weights	DROW	DRIPW
Cultural background	1.21 (1.06–1.39)	1.19 (1.04–1.36)	1.19 (0.98–1.45)	1.19 (1.04–1.36)
Mental health	1.47 (1.28–1.7)	1.27 (1.09–1.48)	1.35 (1.12–1.64)	1.32 (1.08–1.61)
Sexual attraction	1.49 (1.23–1.81)	1.38 (1.13–1.68)	1.37 (1.12–1.68)	1.37 (1.09–1,71)
Sex	1.35 (1.15–1.59)	1.27 (1.08–1.50)	1.27 (1.08–1.49)	1.29 (1.09–1.53)

DRIPW, doubly robust inverse probability weights DROW, doubly robust overlap weights.

## Discussion

Our study provides evidence that sex assigned at birth and sexuality moderate the effect of discrimination on oral health impacts among adolescents. Overall, females had higher proportions of oral health impact compared with males, and sexually diverse youth tended to have worse oral health outcomes compared with non-LGBQ youth. The largest effects of discrimination on oral health impacts were consistently observed among sexually diverse females. To the best of our knowledge, this is the first study to articulate how sex, sexuality, and experience of discrimination converge to affect oral health.

In this study, we adopted a number of different approaches to emulate randomization (i.e., ensure comparability across groups) and mitigate confounding bias. Advantages of using overlap weights over other methods such as inverse probability weighting include stability of weights and exact balancing of covariates on the mean, even for relatively small subgroups (Mlcoch et al. 2019). Overlap weights result in a pseudo population that mimics a highly inclusive pragmatic randomized trial, providing opportunities for applications in the field of real-world science. Inference is drawn in relation to the overlap population (adolescents who share similar characteristics across the measured confounders).

Our findings show a consistent moderating effect of sex and sexuality on the relationship between discrimination and oral health impacts under different estimators. Sexually diverse females were particularly vulnerable to the effects of discrimination on oral health. These results are consistent with evidence from other disciplines showing that sexual minority identities may compound sex disparities in tobacco use, sleep disturbances, self-harm, and suicidality (Delahanty et al. 2019; Butler et al. 2020; Jadva et al. 2023). Gender differences in trajectories of health-related quality of life among adolescents have been observed, with worse outcomes among females (Meade and Dowswell 2016; Berra et al. 2024). Previous studies reported that female adolescents have higher rates of oral health impacts than males do (Vazquez et al. 2015; Sfreddo et al. 2019). These disparities should be contextualized within patriarchal societies, where the sex/gender binary is understood through a rigid heteronormative lens. Patriarchy and compulsory heterosexuality conflate to construct gender hierarchies that sustain traditional gender roles and compound oppression against sexually diverse groups (Schilt and Westbrook 2009).

The minority stress framework posits that increased psychological stress resulting from the experience of stigma is disruptive to a range of important physiological, interpersonal, and cognitive processes that affect the health of sexually diverse youth (Hatzenbuehler and Pachankis 2016). These processes are theorized to mediate the relationship between stigma and psychopathology and offer insights into potential mechanisms by which stigma affects oral health (Hatzenbuehler 2009). It is likely that discrimination may result in more frequent adverse oral health behaviors. Evidence shows that stigma is a contributing factor to explaining inequities in mental health, suicidality, alcohol consumption, and smoking among sexually diverse groups (Delahanty et al. 2019; Zollweg et al. 2023; Li et al. 2024). Proximal minority stressors such as internalized homophobia and fear of rejection may contribute to explaining why sexually diverse adolescents had worse oral health outcomes than non-LGBQ males even in the absence of recent experiences of discrimination.

Policies fostering safe and inclusive schools promote lower rates of victimization, increased school attendance, and improved feelings of safety and connection with the school community among sexually diverse youth (Domínguez-Martínez and Robles 2019). Programs designed to fight intersecting systems of oppression, such as racism, sexism, homophobia, transphobia, and ableism, may provide renewed opportunities to address oral health inequities. At a health care level, findings underscore the need for creating inclusive environments where young people feel comfortable seeking care, disclosing their sexual identity, and discussing health concerns. It is critical that dental practitioners and other allied health providers are equipped to understand principles of intersectionality and embrace trauma-informed care practices (Diop et al. 2021). A considerable proportion of sexually diverse young adults reported discomfort and unfair treatment in oral health settings (Tharp et al. 2022). Building pathways to antiracist, gender-affirming, and LGBTQ+ inclusive standards of care involves the active role of dental practitioners in advocating for marginalized youth both within and outside the health care system.

Findings should be interpreted with caution due to a number of limitations. The weighting methods adopted in this study rely on the assumption of no unmeasured confounding. We were not able to examine the intensity of experiences of discrimination or structural discrimination. Sexuality development is a complex, multifaceted process. Adolescents may experience dimensions of sexual identity, sexual attraction, and sexual behavior in dynamic and fluid ways. In this study, we were able to evaluate only 1 dimension of sexual orientation (sexual attraction). Because information on gender identity was not available, we acknowledge that our study may contribute to the invisibility of gender nonconforming and trans youth in oral health research. Future studies should investigate how compounding forms of discrimination converge to affect the oral health of gender-diverse youth.

## Conclusion

This study highlights the impact of different forms of discrimination on the oral health of adolescents. Findings underscore the moderating role of sex and sexuality in the relationship between discrimination and oral health. Our study sheds light on the intersection of sex, sexuality, and discrimination in shaping oral health outcomes, providing valuable insights for future interventions aimed at addressing oral health inequities among adolescents.

## Author Contributions

G.H. Soares, contributed to conception, design, data analysis and interpretation, draft and critically revised the manuscript; S. Sethi, A. Jessani, L. Jamieson, contributed to conception, data interpretation, critically revised the manuscript. All authors gave final approval and agree to be accountable for all aspects of the work.

## Supplemental Material

sj-docx-1-jdr-10.1177_00220345241310223 – Supplemental material for Discrimination and Oral Health Impact: Moderating Role of Sex and Sexuality Supplemental material, sj-docx-1-jdr-10.1177_00220345241310223 for Discrimination and Oral Health Impact: Moderating Role of Sex and Sexuality by G.H. Soares, S. Sethi, A. Jessani and L. Jamieson in Journal of Dental Research
