# Supplementary material for: Discrimination and Oral Health Impact: Moderating Role of Sex and Sexuality
Source: J Dent Res. 2025 Feb 14;104(5):495–502. doi: 10.1177/00220345241310223 (PMC12000624; doi:10.1177/00220345241310223)
Supplement: sj-docx-1-jdr-10.1177_00220345241310223 – Supplemental material for Discrimination and Oral Health Impact: Moderating Role of Sex and Sexuality [file sj-docx-1-jdr-10.1177_00220345241310223.docx]

**Supplementary files**

Discrimination and oral health impact: moderating role of sex and sexuality

Gustavo Hermes Soares^1^, Sneha Sethi^1^, Abbas Jessani^2^, Lisa Jamieson^1^

Glossary

| Term | Definition |
| --- | --- |
| Gender | Gender is a multidimensional construct that incorporates:   1. gender identity (a core element of a person’s individual identity); 2. gender expression (the ways individuals signal their gender to others); and 3. cultural expectations about social status, characteristics, and behaviours associated with sex traits.   These dimensions of gender do not necessarily align with sex assigned at birth. |
| LGBQ | In this study, LGBQ stands for Lesbian, Gay, Bisexual and Questioning. It refers to individuals attracted to the same sex, both sexes, or unsure about their attraction. Individuals identified as questioning are those unsure about their attraction or still exploring their own sexuality. |
| Non-LGBQ | In this study, non-LGBQ refers to individuals expressing attraction to members of the opposite sex only and adolescents expressing no attractions. |
| Sex assigned at birth | In Western societies, sex is usually assigned at birth by medical professionals as either male or female based on visual inspection of external genitalia. Sex is a multidimensional construct based on a combination of anatomical and physiological characteristics. It includes external genitalia, secondary sex characteristics, gonads, chromosomes, and hormones. |
| Sexual attraction | Sexual attraction is a key dimension of sexual orientation. For the purpose of this study, sexual attraction was defined on the basis of the sex of a person's desired or actual sexual or romantic partners in relation to their own sex. |
| Sexual orientation | Sexual orientation is a multidimensional construct encompassing emotional, romantic, and sexual attraction, identity, and behaviour. |
| Sexuality | Sexuality usually refers to sexual identity, attraction, behaviour and associated psychological, social, and behavioural processes and constructs. In this study, we use the term ‘sexuality’ to refer to the specific domain of sexual attraction. |
| Sexually diverse | Sexually diverse describes individuals who identify as lesbian, gay, bisexual, questioning, or who exhibit attractions and/or behaviours that do not align with heterosexual norms. This definition does not include gender diverse individuals. |

Figure S1. Standardised differences in covariates between adolescents exposed and unexposed to cultural background discrimination before and after application of overlap weights and inverse probability weights for each level of the moderator.


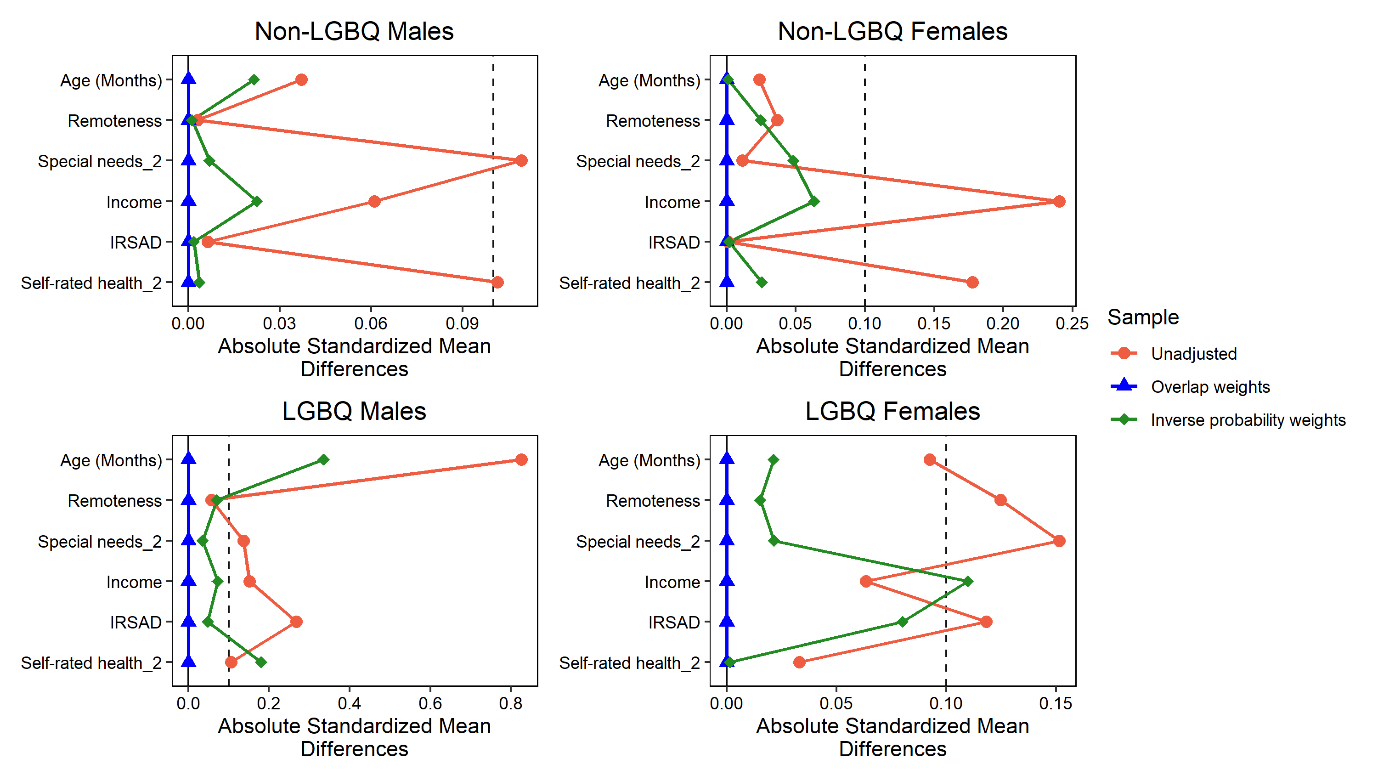


Figure S2. Standardised differences in covariates between adolescents exposed and unexposed to mental health discrimination before and after application of overlap weights and inverse probability weights for each level of the moderator.


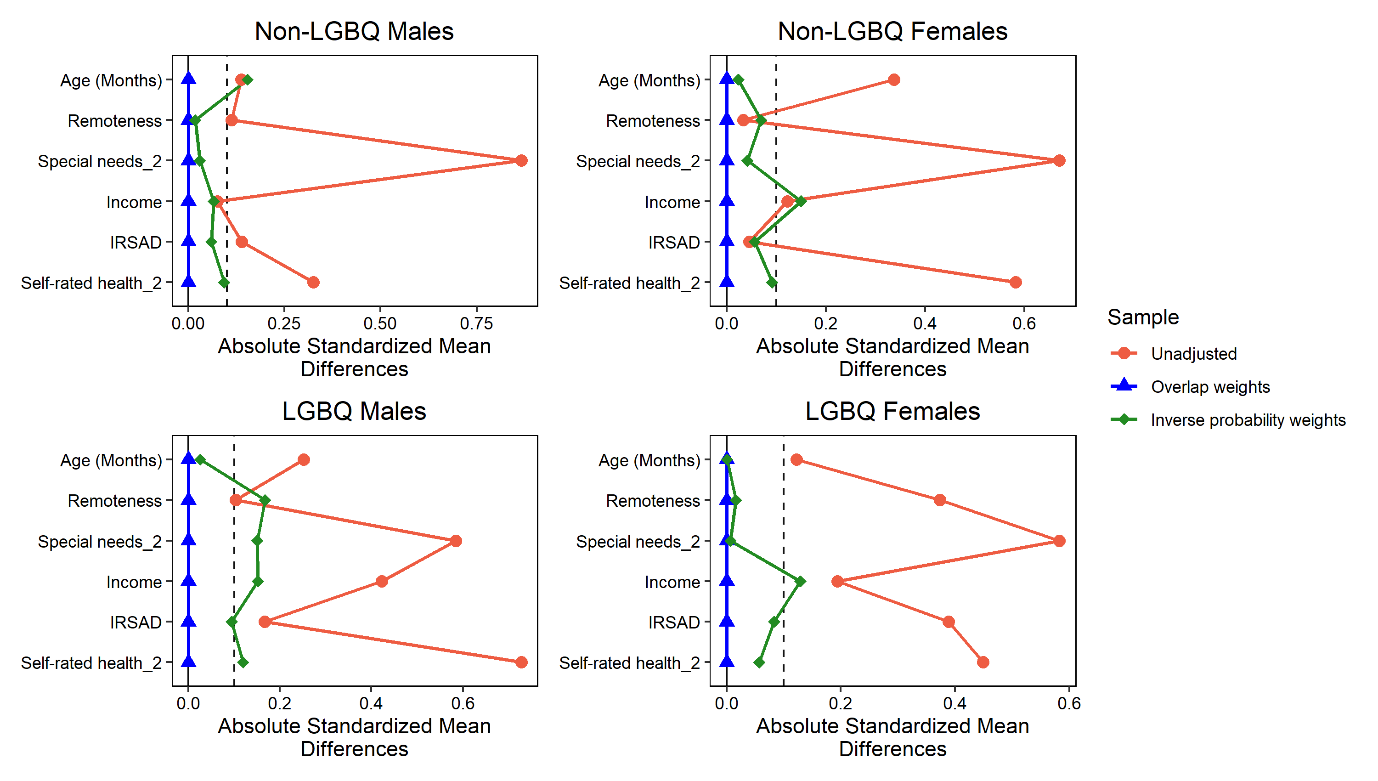


Figure S3. Standardised differences in covariates between adolescents exposed and unexposed to sexual orientation discrimination before and after application of overlap weights and inverse probability weights for each level of the moderator.


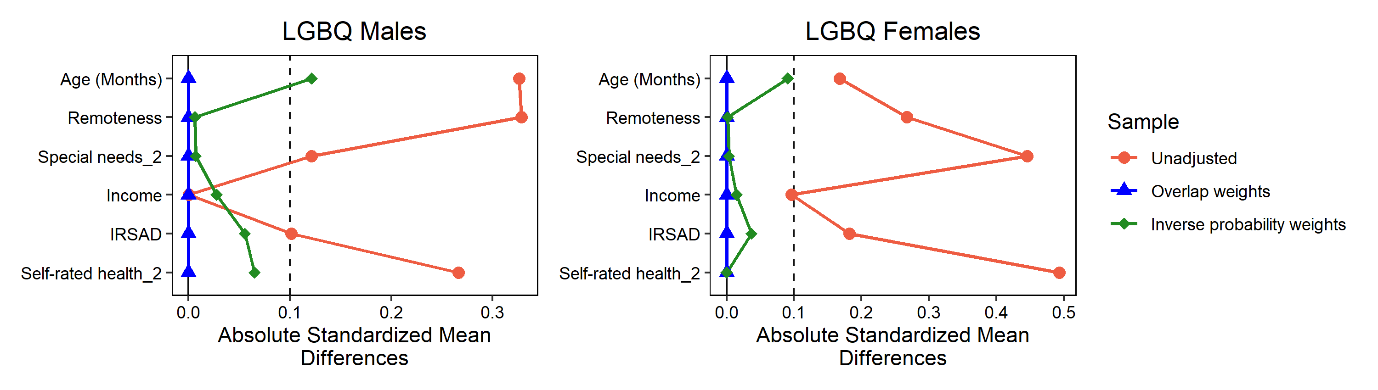


Figure S4. Standardised differences in covariates between adolescents exposed and unexposed to sex discrimination before and after application of overlap weights and inverse probability weights for each level of the moderator.


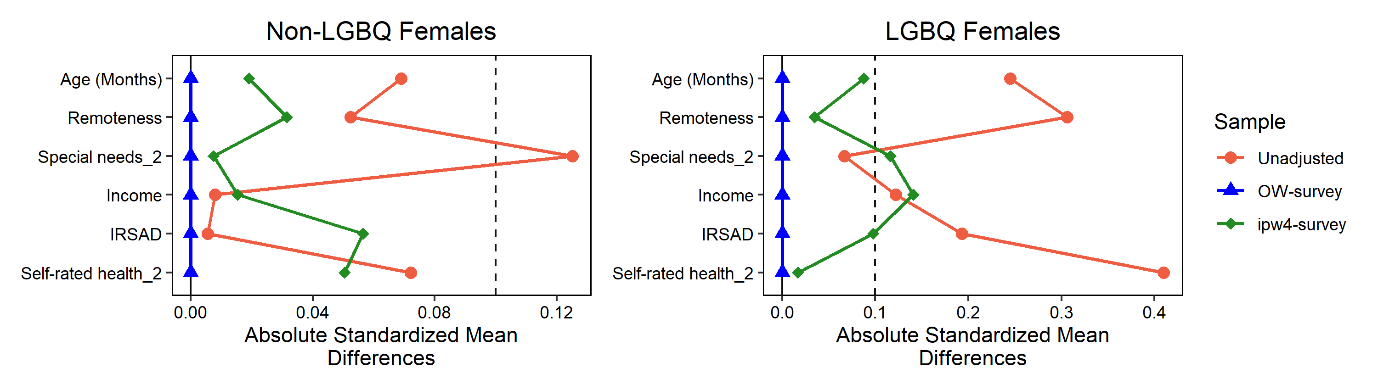


Table S1. Weighted distribution of participants based on experience of discrimination using overlap weights multiplied by survey weights.

|  | Mental health discrimination | | Cultural background discrimination | | Sexual orientation discrimination | | Sex discrimination | |
| --- | --- | --- | --- | --- | --- | --- | --- | --- |
|  | No | Yes | No | Yes | No | Yes | No | Yes |
| Oral health impact | 47.7 (44.3-51.1) | 60.4 (52-68.3) | 42.5 (40.2-44.9) | 50.5 (44.4-56.6) | 53.8 (46.4-61) | 74.1 (61.7-83.5) | 48.5 (45-52.1) | 61.8 (52.1-70.5) |
| Age (months) | 178.97 ±7.72 | 178.97 ±4.23 | 178.3 ±5.2 | 178.3 ±2.9 | 178.5 ±17.9 | 178.5 ±6.9 | 178.2 ±7.7 | 178.2 ±4.6 |
| Self-rated general health |  |  |  |  |  |  |  |  |
| Excellent/Very good | 48 (44.7-51.3) | 48 (39.8-56.3) | 65.5 (63.1-67.8) | 65.5 (59a.4-71.1) | 45.9 (38.8-53.2) | 45.9 (32.5-60) | 57.3 (53.6-60.9) | 57.3 (47.3-66.6) |
| Good/Fair/Poor | 52 (48.7-55.3) | 52 (43.7-60.2) | 34.5 (32.2-36.9) | 34.5 (28.9-40.6) | 54.1 (46.8-61.2) | 54.1 (40-67.5) | 42.7 (39.1-46.4) | 42.7 (33.4-52.7) |
| Special care needs |  |  |  |  |  |  |  |  |
| Yes | 33.7 (30-37.5) | 33.7 (26.5-41.6) | 14.1 (12.3-16) | 14.1 (10.3-18.9) | 22.8 (16.6-30.6) | 22.8 (14.1-34.8) | 19.2 (16.1-22.7) | 19.2 (12.2-29) |
| No | 66.3 (62.5-70) | 66.3 (58.4-73.5) | 85.9 (84-87.7) | 85.9 (81.1-89.7) | 77.2 (69.4-83.4) | 77.2 (65.2-85.9) | 80.8 (77.3-83.9) | 80.8 (71-87.8) |
| Remoteness |  |  |  |  |  |  |  |  |
| Major Cities | 60.8 (57.7-63.9) | 60.8 (52.5-68.5) | 67 (65-68.9) | 67 (61.2-72.4) | 63.4 (55.7-70.4) | 63.4 (50.2-74.8) | 68.9 (65.9-71.8) | 68.9 (59.6-76.9) |
| Regional | 39.2 (36.1-42.3) | 39.2 (31.5-47.5) | 33 (31.1-35) | 33 (27.6-38.8) | 36.6 (29.6-44.3) | 36.6 (25.2-49.8) | 31.1 (28.2-34.1) | 31.1 (23.1-40.4) |
| Weekly income (AU$) | 1080.2 ±1462.2 | 1080.2 ±801.6 | 989.9 ±823.4 | 989.9 ±499.4 | 1177.4 ±1084.7 | 1177.4±1268.7 | 1130.4 ±481.3 | 1130.4 ±627.9 |
| SEIFA | 996.2 ±141.5 | 996.2 ±82.7 | 1005.3 ±96.5 | 1005.3 ±90.7 | 999.41 ±116.4 | 999.41 ±93.9 | 1010.6 ±45.6 | 1010.6 ±63.4 |

|  | **Straight** | | | | **LGBQ** | | | |
| --- | --- | --- | --- | --- | --- | --- | --- | --- |
|  | **Male** | | **Female** | | **Male** | | **Female** | |
| Mental health discrimination | No | Yes | No | Yes | No | Yes | No | Yes |
| Oral health impact | 39.5 (34.7-44.5) | 44 (29.9-59.1) | 48.8 (43.9-53.7) | 65.5 (53.2-76.1) | 52.4 (36.1-68.2) | 39.5 (11-77.6) | 58.8 (48.9-68) | 80.4 (63.7-90.5) |
| Age (months) | 178.80 ±8.32 | 178.80 ±4.04 | 179.46 ±7.25 | 179.46 ±4.29 | 179.42 ±6.81 | 179.42 ±2.60 | 177.89 ±5.22 | 177.89 ±3.83 |
| Self-rated general health |  |  |  |  |  |  |  |  |
| Excellent/Very good | 59.1 (53.7-64.4) | 59.1 (43.8-72.9) | 43.8 (39.3-48.5) | 43.8 (32.6-55.7) | 27.2 (17.6-39.6) | 27.2 (6.4-67.2) | 42.5 (33.5-52) | 42.5 (25.3-61.6) |
| Good/Fair/Poor | 40.9 (35.6-46.3) | 40.9 (27.1-56.2) | 56.2 (51.5-60.7) | 56.2 (44.3-67.4) | 72.8 (60.4-82.4) | 72.8 (32.8-93.6) | 57.5 (48-66.5) | 57.5 (38.4-74.7) |
| Special care needs |  |  |  |  |  |  |  |  |
| Yes | 41.1 (35.5-46.9) | 41.1 (27.5-56.3) | 30.5 (25.3-36.3) | 30.5 (20.8-42.4) | 32.9 (17.4-53.4) | 32.9 (9.6-69.5) | 28.6 (19.5-39.9) | 28.6 (16.1-45.5) |
| No | 58.9 (53.1-64.5) | 58.9 (43.7-72.5) | 69.5 (63.7-74.7) | 69.5 (57.6-79.2) | 67.1 (46.6-82.6) | 67.1 (30.5-90.4) | 71.4 (60.1-80.5) | 71.4 (54.5-83.9) |
| Remoteness |  |  |  |  |  |  |  |  |
| Major Cities | 53.8 (48.8-58.7) | 53.8 (38.5-68.4) | 65.2 (60.6-69.5) | 65.2 (53.1-75.5) | 63.4 (46.4-77.6) | 63.4 (24.1-90.4) | 61.6 (51.4-70.9) | 61.6 (44-76.6) |
| Regional | 46.2 (41.3-51.2) | 46.2 (31.6-61.5) | 34.8 (30.5-39.4) | 34.8 (24.5-46.9) | 36.6 (22.4-53.6) | 36.6 (9.6-75.9) | 38.4 (29.1-48.6) | 38.4 (23.4-56) |
| Weekly income (AU$) | 1063 ±1199.6 | 1063 ±599.1 | 1034.5 ±1107.9 | 1034.5 ±772.1 | 1975.6 ±4330.7 | 1975.6 ±2000.3 | 1077.4 ±963.6 | 1077.4 ±831.6 |
| SEIFA | 991.35 ±127.34 | 991.35 ±80.88 | 1002.76 ±121.61 | 1002.76 ±77.72 | 1005.8 ±109.1 | 1005.8 ±82.4 | 985.9 ±82.4 | 985.9 ±94.7 |

Table S2. Weighted distribution of participants based on experience of cultural background discrimination for each level of the moderator using overlap weights multiplied by survey weights.

Table S3. Weighted distribution of participants based on experience of mental health discrimination for each level of the moderator using overlap weights multiplied by survey weights.

|  | **Straight** | | | | **LGBQ** | | | |
| --- | --- | --- | --- | --- | --- | --- | --- | --- |
|  | **Male** | | **Female** | | **Male** | | **Female** | |
| Cultural background discrimination | No | Yes | No | Yes | No | Yes | No | Yes |
| Oral health impact | 36.9 (33.8-40.2) | 44.9 (36.4-53.6) | 45.7 (41.9-49.5) | 53.1 (43.1-62.8) | 41.1 (28.4-55.1) | 51.4 (26.9-75.2) | 61.1 (52.7-68.8) | 71 (50.4-85.5) |
| Age (months) | 178.5 ±4.9 | 178.5 ±3.9 | 178.4 ±4.9 | 178.4 ±4.4 | 176 ±4.7 | 176 ±2.9 | 178.1 ±4.7 | 178.13 ±4.3 |
| Self-rated general health |  |  |  |  |  |  |  |  |
| Excellent/Very good | 71.8 (68.5-74.8) | 71.8 (63.4-78.8) | 60.4 (56.4-64.2) | 60.4 (50-69.9) | 69.5 (57.1-79.6) | 69.5 (44.2-86.8) | 50.4 (42-58.8) | 50.4 (30.5-70.1) |
| Good/Fair/Poor | 28.2 (25.2-31.5) | 28.2 (21.2-36.6) | 39.6 (35.8-43.6) | 39.6 (30.1-50) | 30.5 (20.4-42.9) | 30.5 (13.2-55.8) | 49.6 (41.2-58) | 49.6 (29.9-69.5) |
| Special care needs |  |  |  |  |  |  |  |  |
| Yes | 12.9 (10.6-15.7) | 12.9 (8.4-19.3) | 13.2 (10.8-16.2) | 13.2 (7.2-23) | 21.1 (11.5-35.7) | 21.1 (7.7-46.3) | 20.5 (14.2-28.6) | 20.5 (8.7-40.9) |
| No | 87.1 (84.3-89.4) | 87.1 (80.7-91.6) | 86.8 (83.8-89.2) | 86.8 (77-92.8) | 78.9 (64.3-88.5) | 78.9 (53.7-92.3) | 79.5 (71.4-85.8) | 79.5 (59.1-91.3) |
| Remoteness |  |  |  |  |  |  |  |  |
| Major Cities | 66.8 (63.9-69.7) | 66.8 (58.3-74.4) | 67.6 (64.3-70.8) | 67.6 (57.9-76.1) | 69.5 (55.4-80.7) | 69.5 (44.2-86.8) | 64 (55.3-71.9) | 64 (42.2-81.3) |
| Regional | 33.2 (30.3-36.1) | 33.2 (25.6-41.7) | 32.4 (29.2-35.7) | 32.4 (23.9-42.1) | 30.5 (19.3-44.6) | 30.5 (13.2-55.8) | 36 (28.1-44.7) | 36 (18.7-57.8) |
| Weekly income (AU$) | 991.9 ±775.2 | 991.9 ±711.7 | 958.7 ±727.1 | 958.7 ±606.6 | 1140.5 ±1298.8 | 1140.5 ±844.3 | 1040.9 ±789.9 | 1040.9 ±855.3 |
| SEIFA | 1007.5 ±88.9 | 1007.5 ±87.6 | 1006.3 ±101.6 | 1006.3 ±83.9 | 1017.5 ±118.8 | 1017.5 ±118.8 | 983.2 ±114.8 | 983.2 ±109.1 |

Table S4. Weighted distribution of participants based on experience of sexual orientation discrimination for each level of the moderator using overlap weights multiplied by survey weights.

|  | **LGBQ** | | | |
| --- | --- | --- | --- | --- |
|  | **Male** | | **Female** | |
| Sexual orientation discrimination | No | Yes | No | Yes |
| Oral health impact | 46.1 (33.7-58.9) | 55.4 (33.7-75.3) | 57.1 (48.1-65.7) | 82.1 (67.7-91) |
| Age (months) | 178.9 ±17.9 | 178.9 ±6.9 | 178.3 ±4.7 | 178.3 ±4.7 |
| Self-rated general health |  |  |  |  |
| Excellent/Very good | 55.3 (42.3-67.7) | 55.3 (33.6-75.2) | 41.9 (33.7-50.5) | 41.9 (25.6-60.1) |
| Good/Fair/Poor | 44.7 (32.3-57.7) | 44.7 (24.8-66.4) | 58.1 (49.5-66.3) | 58.1 (39.9-74.4) |
| Special care needs |  |  |  |  |
| Yes | 20.1 (11.8-32.2) | 20.1 (8.1-42.1) | 24 (16.2-34) | 24 (13.4-39.2) |
| No | 79.9 (67.8-88.2) | 79.9 (57.9-91.9) | 76 (66-83.8) | 76 (60.8-86.6) |
| Remoteness |  |  |  |  |
| Major Cities | 51.6 (38.9-64.1) | 51.6 (30.2-72.5) | 68.4 (59.1-76.5) | 68.4 (52.9-80.7) |
| Regional | 48.4 (35.9-61.1) | 48.4 (27.5-69.8) | 31.6 (23.5-40.9) | 31.6 (19.3-47.1) |
| Weekly income (AU$) | 1243.7 ±1370.9 | 1243.7 ±1184.3 | 1148.9 ±940.6 | 1148.9 ±1292.4 |
| SEIFA | 1005.6 ±105.6 | 1005.6 ±81.1 | 996.7 ±120.4 | 996.7 ±98 |

Table S5. Weighted distribution of participants based on experience of sexual orientation discrimination for each level of the moderator using overlap weights multiplied by survey weights.

|  | **Females** | | | |
| --- | --- | --- | --- | --- |
|  | **Non-LGBQ** | | **LGBQ** | |
| Sex discrimination | No | Yes | No | Yes |
| Oral health impact | 45 (41.4-48.7) | 59.3 (47.8-69.8) | 59.1 (50.2-67.5) | 69.3 (51.5-82.8) |
| Age (months) | 178.5 ±17.9 | 178.5 ±6.9 | 177.2 ±4.7 | 177.2 ±4.7 |
| Self-rated general health |  |  |  |  |
| Excellent/Very good | 63.8 (60-67.3) | 63.8 (51.4-74.5) | 37.5 (29.8-45.8) | 37.5 (22.9-54.7) |
| Good/Fair/Poor | 36.2 (32.7-40) | 36.2 (25.5-48.6) | 62.5 (54.2-70.2) | 62.5 (45.3-77.1) |
| Special care needs |  |  |  |  |
| Yes | 19.2 (16-22.9) | 19.2 (10.9-31.5) | 19.3 (12.4-28.7) | 19.3 (9.2-36) |
| No | 80.8 (77.1-84) | 80.8 (68.5-89.1) | 80.7 (71.3-87.6) | 80.7 (64-90.8) |
| Remoteness |  |  |  |  |
| Major Cities | 65.8 (62.6-69) | 65.8 (54.6-75.6) | 78.2 (71-84.1) | 78.2 (60.1-89.6) |
| Regional | 34.2 (31-37.4) | 34.2 (24.4-45.4) | 21.8 (15.9-29) | 21.8 (10.4-39.9) |
| Weekly income (AU$) | 1125.7 ±287.2 | 1125.7 ±361.6 | 1144.7 ±971.9 | 1144.7 ±1340.3 |
| SEIFA | 1008.8 ±26.3 | 1008.8 ±38.4 | 1016.2 ±98.3 | 1016.2 ±125.3 |

Table S6. Estimated effects of discrimination on prevalence of oral health impact by sexual orientation and sex using doubly robust estimators.

|  |  | **Non-LGBQ** | | **LGBQ** | |
| --- | --- | --- | --- | --- | --- |
| **Discrimination** | **Sample** | **Males** | **Females** | **Males** | **Females** |
|  | **PR (95% CI)** | **PR (95% CI)** | **PR (95% CI)** | **PR (95% CI)** | **PR (95% CI)** |
| **DROW** |  |  |  |  |  |
| Cultural background |  |  |  |  |  |
| Absent | 1 | 1 | 1.21 (1.07-1.36) | 1.11 (0.79-1.57) | 1.56 (1.33-1.83) |
| Present | 1.19 (0.98-1.45) | 1.22 (0.99-1.5) | 1.4 (1.11-1.71) | 1.4 (0.64-2.11) | 1.82 (1.31-2.29) |
| Mental health |  |  |  |  |  |
| Absent | 1 | 1 | 1.19 (1.01-1.4) | 1.25 (0.78-1.58) | 1.46 (1.17-1.78) |
| Present | 1.35 (1.12-1.64) | 1.11 (0.78-1.58) | 1.6 (1.28-1.97) | 0.95 (0.34-2.64) | 1.99 (1.56-2.47) |
| Sexual orientation |  |  |  |  |  |
| Absent | 1 |  |  | 1 | 1.24 (0.9-1.71) |
| Present | 1.37 (1.12-1.68) |  |  | 1.2 (0.75-1.92) | 1.77 (1.31-2.54) |
| Sex |  |  |  |  |  |
| Absent | 1 |  | 1 |  | 1.32 (1.1-1.58) |
| Present | 1.27 (1.08-1.49) |  | 1.31 (1.08-1.6) |  | 1.54 (1.16-1.95) |
| **DRIPW** |  |  |  |  |  |
| Cultural background |  |  |  |  |  |
| Absent | 1 | 1 | 1.21 (1.07-1.36) | 1.15 (0.87-1.53) | 1.51 (1.29-1.79) |
| Present | 1.19 (1.04-1.36) | 1.21 (0.98-1.50) | 1.41 (1.13-1.72) | 1.38 (0.59-2.24) | 1.74 (1.25-2.22) |
| Mental health |  |  |  |  |  |
| Absent | 1 | 1 | 1.17 (1.04-1.31) | 1.20 (0.93-1.55) | 1.43 (1.35-2.18) |
| Present | 1.32 (1.08-1.62) | 1.14 (0.76-1.71) | 1.75 (1.35-2.18) | 1.33 (0.51-3.44) | 1.94 (1.42-2.42) |
| Sexual orientation |  |  |  |  |  |
| Absent | 1 |  |  | 1 | 1.24 (0.91-1.70) |
| Present | 1.37 (1.10-1.71) |  |  | 1.07 (0.61-1.86) | 1.85 (1.34-2.59) |
| Sex |  |  |  |  |  |
| Absent | 1 |  | 1 |  | 1.31 (1.11-1.54) |
| Present | 1.29 (1.09-1.54) |  | 1.31 (1.07-1.60) |  | 1.60 (1.17-2.03) |
|  |  |  |  |  |  |
